# The flavonoid-rich ethanolic extract from the green cocoon shell of silkworm has excellent antioxidation, glucosidase inhibition, and cell protective effects *in vitro*

**DOI:** 10.29219/fnr.v64.1637

**Published:** 2020-08-14

**Authors:** Hai-Yan Wang, Jin-Ge Zhao, Yu-Qing Zhang

**Affiliations:** Silk Biotechnology Laboratory, School of Biology and Basic Medical Sciences, Soochow University, Dushuhu Higher Edu. Town, Suzhou, P R China

**Keywords:** sericin, ethanolic extract, antioxidation, glucosidase inhibition, cell protective effect

## Abstract

The green cocoon shell of a novel variety of silkworm, *Bombyx mori,* is rich in two types of quercetin and kaempferol flavonoids. The aim of this study was to identify these flavonoids in the ethanolic extract (EE) from green cocoons and develop EE applications in healthy foods. The experimental results indicated that the amount of total amino acids in EE was 27.06%. The flavonoids in EE are presented in quercetin and kaempferol glycosides. The total amount of the two aglycones was 33.42 ± 0.08 mg/g. The IC_50_ values of 2,2-diphenyl-1-picrylhydrazyl (DPPH), 1,2’-azino-bis (3-ethylbenzthiazoline-6-sulphonicacid) (ABTS), and hydroxyl radical scavenging abilities were 296.95 ± 13.24 μg/mL, 94.31 ± 9.13 μg/mL, and 9.21 ± 0.15 mg/mL, respectively. The IC_50_ values of the inhibitory activities of α-amylase and α-glucosidase were 37.57 ± 6.45 μg/mL and 212.69 ± 22.94 μg/mL, respectively. EE also reduced the level of reactive oxygen species (ROS) and oxidative stress in L02 cells induced by high glucose levels. It also effectively decreased the content of 8-hydroxyl deoxyguanosine (8-OHdG), nuclear factor κB (NF-κB), and tumour necrosis factor alpha (TNF-α) in cells with a good dose effect. These results clearly indicated that the flavonoid-rich EE with excellent antioxidant and glucosidase inhibition abilities significantly reduced the damage to cells caused by oxidative stress and inflammatory reactions. It is suggested that EE might serve as useful functional foods for the treatment of related diseases induced by oxidative stress such as diabetes mellitus.

## Popular scientific summary

Here, we choose a new green cocoon variety (caoyuan × shenyun) which combines the advantages of Daizo cocoon and normal white cocoon as material to identify the flavonoids in the ethanolic extract from green cocoons and develop the applications of ethanolic extract in healthy foods.

The cocoon shell from the silkworm *B. mori* is composed of 70% silk fibroin fibre surrounded by 25% sericin and the other 5% comprising non-sericin components (1). The 5% non-sericin ‘impurities’ consist of pigment, wax, carbohydrates, flavonoids, and free amino acids, which have high scientific and commercial value.

Sericin is a type of colloidal spherical protein that acts as an adhesive to join two fibroin filaments to form a solid ellipsoidal cocoon shell (2). Both *in vitro* and *in vivo* studies have demonstrated that sericin, which is immunologically inert (3, 4), has ultraviolet radiation resistant (5, 6), skin whitening (7), antioxidant (8), and anticancer activities. Food and Drug Administration (FDA) has already included sericin and its derivatives in the ‘Generally Recognized as Safe – GRAS’ list (9). As a dietary food, the main characteristic is its antioxidant function. Sericin can be added to bread and drinks or other kinds of food (10) and is demonstrated to be useful for treatment of constipation, and can suppress the incidence and number of colon tumors (11–13). Therefore, sericin is safe and widely applicable in food and cosmetic industries and applications in biomedicine, such as cryopreservation, wound healing, various metabolic effects in organic systems, and as a vehicle for drug delivery (14–16).

It is worth noting that studies mentioned above often use mixtures of sericin peptides of wide-ranging molecular masses as starting materials. Few experiments were carried out using pure sericin samples that were free of non-sericin components. Our previous study compared the bioactivities of pure sericin with those of non-sericin components from different cocoons. The results demonstrated that the ethanol extracts, which mainly contained flavonoids and free amino acids, possessed greater inhibiting activities of tyrosinase and scavenging activities of 2,2-diphenyl-1-picrylhydrazyl (DPPH) free radicals. In addition, the ethanol extracts showed strong inhibition of α-glucosidase, while the purified sericin had no such activity. In addition, the ethanol extracts from Daizo cocoons contained more flavonoids (17).

Flavonoids have been found as pigments in the cocoon shells of some silkworm species (18–20). The separation, purification, identification, and bioactivities of flavonols in cocoons have attracted increasing attention. The yellow green Irodori cocoon harvested in Saitama Prefecture is a flavonol-rich cocoon. After extraction in 80% methanol and boiling in hot water to separate sericin and fibroin fractions, the flavonol content in the three fractions, that is, the free flavonol fraction, sericin fraction, and fibroin fraction, were found to be 2,227, 132, and 226 μg/g cocoon shells, respectively (21). Kurioka et al. isolated two kaempferol glycosides (kaempferol 7-O-*β*-D-glucoside and kaempferol 5-O-*β*-D-glucoside) and three quercetin glycosides (quercetin 4’-O-*β*-D-glucoside, quercetin 5-O-*β*-D-glucoside and quercetin 7-O-*β*-D-glucoside), along with their aglycones, kaempferol and quercetin from an ethanolic extract (EE) of Sasamayu cocoon shells (22). Even though mulberry leaves are the silkworm’s only food, scientists have found that there are flavonoid glucosides in cocoons that are not present in mulberry leaves. Therefore, the flavonoid glucosides extracted from cocoons are not only from the mulberry leaves, and some may be metabolites produced by the silkworm (23). In addition to the flavonoid aglycones, kaempferol and quercetin, two flavonoids containing the L-proline moiety were found in cocoons, which is the first time that flavonoids with an amino acid moiety have been found as naturally occurring compounds. However, these compounds are also not found in the mulberry leaves, suggesting that these flavonoids are metabolites of the silkworm (24).

Sericin has been proved to have antidiabetic activity (25). Hyperglycaemia is the classic symptoms of diabetes. Persistent hyperglycaemia will cause the excessive production of ROS and induce oxidative stress, which results in DNA damage and a cascade of other reactions including inflammation. NF-κB, a major target of ROS, can be activated by excessive ROS and the activation of NF-κB-dependent genes triggers several pathways, that is, the production of proinflammatory cytokine tumour necrosis factor alpha (TNF-α) (26).

The liver is the main target of insulin and the key organ for glucose metabolism. Besides, the liver is also the major organ prone to be damaged by oxidative stress. Here, for a better study of the antidiabetic activity of EE, a new green cocoon variety (caoyuan × shenyun) which combines the advantages of Daizo cocoon and normal white cocoon was chosen as the material. The antioxidation, glucosidase inhibition, and protective effects on L02 cells of EE were measured *in vitro*.

## Materials and methods

### Chemicals

DPPH, α-amylase, α-glucosidase, and 4-Nitrophenyl-β-D-lucopyranoside substrate were purchased from Sigma (America). The amylose was purchased from Shanghai green leaf Biotechnology Co., Ltd. (Shanghai, China). 1, 2’-Azino-bis(3-ethylbenzthiazoline-6-sulphonicacid) (ABTS) was purchased from Shanghai Aladdin Biochemical Polytron Technologies Inc. Kaempferol standard products were purchased from Yunnan Xili Biotechnology Limited by Share Ltd. (KunMing, China). Quercetin was purchased from Shanghai Chemical Reagent Co. Ltd. (Shanghai, China). All of the other chemicals and solvents used were of analytical grade except acetonitrile and methanol (HPLC grade).

### Preparation of EE from the cocoon layer

Green cocoons (Caoyuan × Shenyun), a new flavonoid-rich cocoon of silkworm, were obtained from the Soochow University Sericulture Institute (Suzhou, China). The green cocoons were collected in the Spring of 2017. This green cocoon of the silkworm bred by Professor Yu Xiao-Hua from Soochow University is a hybrid between Daizo and the commercially common silkworm hybrid (Jingsong × Haoyue) (27).

Sericin was achieved after boiling (20 min × 2) in a 0.025% calcium hydroxide solution. The alkaline degumming water containing sericin was neutralized by sulphuric acid. Then, a crude sericin solution was obtained after centrifugation. After filtration, the resulting crude sericin solution was mixed with ethanol to give a final concentration of 70%. The precipitate was washed repeatedly with a 70% ethanol solution. The supernatant was collected, evaporated, and freeze-dried. The resulting powdered extract was further used for the following experiments.

### Analysis of amino acid and flavonoids in EE

EE and sericin were hydrolysed in 6 mol/L HCL at 110 °C for 24 h. Then, the amino acid was measured by an automatic amino acid analyser.

Liquid chromatography-tandem mass chromatography (LC-MS/MS) analyses were performed using an Ultra High Pressure Liquid Chromatography (UHPLC) system (1290, Agilent Technologies) with a UHPLC BEH Amide column (1.7 μm 2.1 × 100 mm, Waters) coupled to TripleTOF 5600 (Q-TOF, AB Sciex). The mobile phase consisted of 25 mM NH_4_OAc and 25 mM NH_4_OH in water (pH = 9.75) (A), and acetonitrile (B) was carried with an elution gradient as follows: 0 min, 95% B; 7 min, 65% B; 9 min, 40% B; 9.1 min, 95% B; 12 min, 95% B, which was delivered at 0.5 mL min^-1^. The injection volume was 4 μL. The Triple TOF mass spectrometer was used for its ability to acquire MS/MS spectra in an information-dependent basis (IDA) during the LC/MS experiment. In this mode, the acquisition software (Analyst TF 1.7, AB Sciex) continuously evaluates the full scan survey MS data as it collects and triggers the acquisition of MS/MS spectra depending on preselected criteria. In each cycle, 12 precursor ions with an intensity greater than 100 were chosen for fragmentation at a collision energy (CE) of 30 V (15 MS/MS events with a product ion accumulation time of 50 msec each). ESI source conditions were set as follows: ion source gas 1 as 60 psi, ion source gas 2 as 60 psi, curtain gas as 35 psi, source temperature 650°C, ion spray voltage floating (ISVF) 5,000 or −4,000 V in positive or negative modes, respectively.

MS raw data files were converted to the mzXML format using ProteoWizard and processed by R package XCMS (version 3.2). The pre-processing results generated a data matrix that consisted of the retention time (RT), mass-to-charge ratio (m/z) values, and peak intensity. The R program package was used for peak annotation after XCMS data processing. An in-house MS2 database was applied for metabolite identification.

### Assay of total flavonoids by the hydrolysis-assisted method

A previously reported method was slightly modified for hydrolysis and high-performance liquid chromatography (HPLC) analysis (28). EE was dissolved in ethanol–hydrochloric acid–water (7/2/1, v/v/v) solution and at 75°C by ultrasound (40 kHz) for 60 min. Then, the supernatant was used in the following analysis.

HPLC was conducted by using a Shimadzu HPLC system (Shimadzu, Japan), which consisted of a pump (LC-20AT), diode array detector (DAD, SPD-M20A), C18 column (Agilent 250 × 4.6 mm), and LC-solution system manager program. The mobile phase comprised methyl alcohol–water–acetic acid in a ratio of 500/500/0.4 (v/v/v). The flow rate was 1 mL/min, and the eluate absorbance was monitored at 370 nm using a scanning range of 200–600 nm. The injection volume of the extract was 10 μL.

## Antioxidant assays

### 1,1-diphenyl–2 picrylhydrazyl (DPPH) free radical scavenging ability

The DPPH radical scavenging activity of EE was measured using the method proposed by Zhao (18).

### 1,2’-azino-bis(3-ethylbenzthiazoline-6-sulphonicacid) radical scavenging ability

The ABTS radical scavenging ability of EE was measured using the method of Olabiyi (29).

### Hydroxyl radical (HO·) scavenging activity

The scavenging activity of HO·was determined according to a previous method (30).

### α-Amylase inhibition assay

The method described by Fuwa (31) based on the starch-iodine test was adopted for evaluating α-amylase inhibition.

### α-Glucosidase inhibition assay

The α-glucosidase inhibition was measured using the method proposed by Tibbot (32).

### Cell culture

L02 hepatocytes were obtained from the Chinese Academy of Sciences (China) and were cultured in Roswell Park Memorial Institute (RPMI) 1640 media supplemented with 10% fetal bovine serum (FBS) and 0.5% antibiotic–antimycotic (penicillin–streptomycin–amphotericin B mix) at 37°C in a humidified atmosphere containing 5% CO_2_. Cells at 80% confluence were used for all experiments.

### Cell viability assay

The cytotoxicity of the extract was determined using the CCK8 assay (Beyotime Biotechnology Co. Ltd. Shanghai, China). The cells were trypsinized and seeded in 96-well plates (7 × 10^3^ cells per well). After attaining 80% confluence, the cells were treated with different concentrations of EE for 24 h. After incubation, the cells were treated with CCK8 reagent (10 µL/well) and incubated for 1 h. Then, the absorbance was measured at 450 nm.

### Determination of the effect of EE on the viability of L02 cells cultured in high glucose

The cells were trypsinized and seeded in 96-well plates (7 × 10^3^ cells per well). Cells in the normal group (normal medium), model group (normal medium + 30 mmol/L glucose), and EE-treated groups (normal medium + 30 mmol/L glucose + different concentrations of EE) were cultured for 24 h. Then, the CCK8 assay was used to estimate the viability of L02 cells.

### Determination of the effect of EE on the content of ROS in L02 cells cultured in high glucose

The cells were trypsinized and seeded in 96-well plates (7 × 10^3^ cells per well). Cells in the normal group (normal medium), model group (normal medium + 30 mmol/L glucose), and EE-treated groups (normal medium + 30 mmol/L glucose + different concentrations of EE) were cultured for 24 h. The effect of EE on intracellular ROS levels was measured using 2’,7’-Dichlorofluorescin diacetate (DCFH-DA) as per the manufacturer’s instructions (Nanjing Jiancheng Bioengineering Institute, Nanjing, China.).

### Quantitative estimation of cytokine levels by ELISA

The cells were trypsinized and seeded in 96-well plates (7 × 10^3^ cells per well). Cells in the normal group (normal medium), model group (normal medium + 30 mmol/L glucose), and EE-treated groups (normal medium + 30 mmol/L glucose + different concentrations of EE) were cultured for 24 h. The cells were collected, and TNF-α, NF-κB, and 8-OHdG levels were evaluated using ELISA kits (Shanghai Yuanye Biotechnology Co., Ltd. Shanghai, China).

### Statistics

The experimental data are expressed as the mean ± standard deviation (SD). Significant differences between two sets of data were assessed using one-way ANOVA (Origin 8.5 version). A value of *P* < 0.05 was considered statistically significant.

## Results

### The amino acid and flavonoids in EE

Sericin is a spherical protein composed of 18 amino acids. The amount of total amino acids in the EE from the sericin layer was 27.06%, and the composition was similar to sericin. Serine, proline, aspartic acid, and glycine were the most abundant amino acids, accounting for 13.91, 12.88, 12, and 8.19% of the total amino acid in EE, respectively. Human essential amino acids account for 27.07% of total amino acids (Table 1).

**Table 1:** The amino acid compositions of EE and sericin

Amino acid	EE	EE	Sericin*
ASP	3.25	12.00	3.27
THR	1.63	3.03	6.71
SER	3.76	13.91	20.52
GLU	1.86	6.86	5.45
GLY	2.22	8.19	22.89
ALA	1.38	5.10	12.25
CYS	0.12	0.44	<0.22
VAL	1.72	6.36	3.99
MET	0.23	0.85	0.21
ILE	0.70	2.58	1.30
LEU	1.64	6.07	1.58
TYR	1.74	6.44	6.02
PHE	0.70	2.58	1.45
HIS	1.12	4.14	0.10
LYS	0.70	2.60	0.91
ARG	0.80	2.96	7.28
PRO	3.59	12.88	6.06
Total	27.06^#^	100	100

EE, ethanolic extract.

* The proportion of total amino acids in sericin;

# The proportion of total amino acids in EE.

**Fig. 1:**
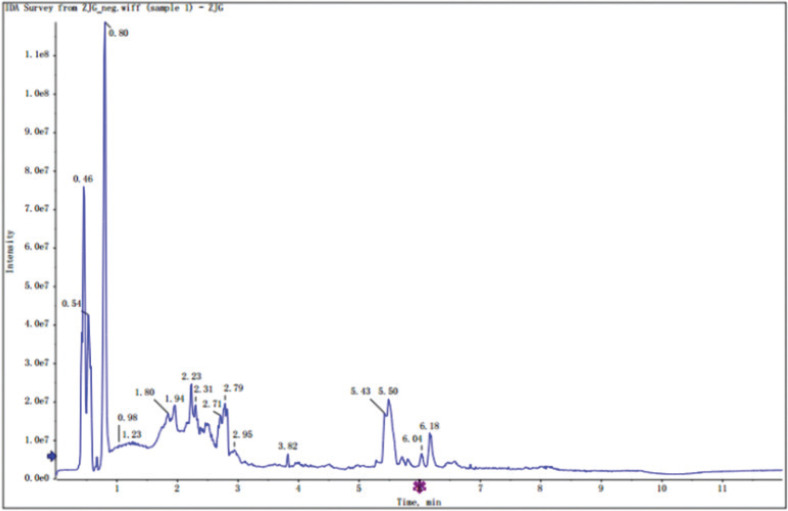
Total ion chromatogram in negative ion mode.

**Fig. 2:**
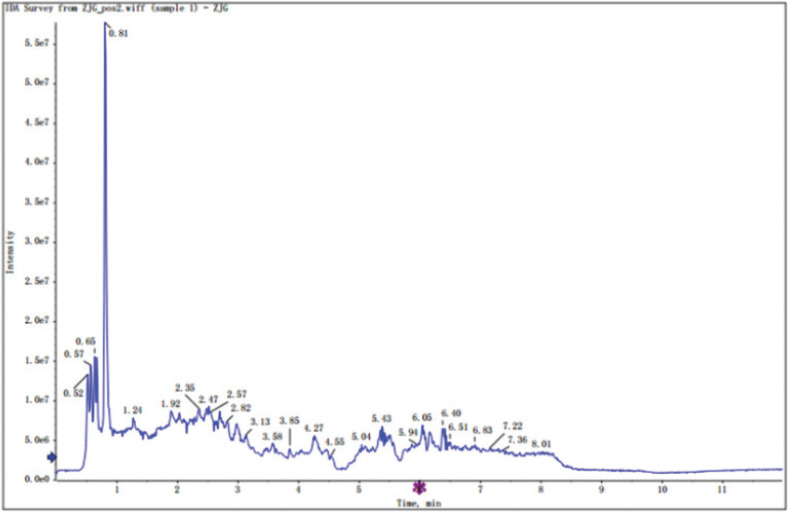
Total ion chromatogram in positive ion mode.

Flavonoids are commonly present in their glycosylated forms and mostly exist as quercetin and kaempferol glycosides in the sericin layers of green cocoons. Rutin, astragalin, quercetin, kaempferol, kaempferol-5-O-glucoside, quercetin-3-O-galactopyranoside, quercetin-3-O-rutinoside, quercetin-3-O-glucoside, quercetin-4’,7-2-O-glucoside were identified by UHPLC-QTOF-MS using R program package and internal MS2 data (total ion chromatogram, Figs. 1 and 2).

**Fig. 3:**
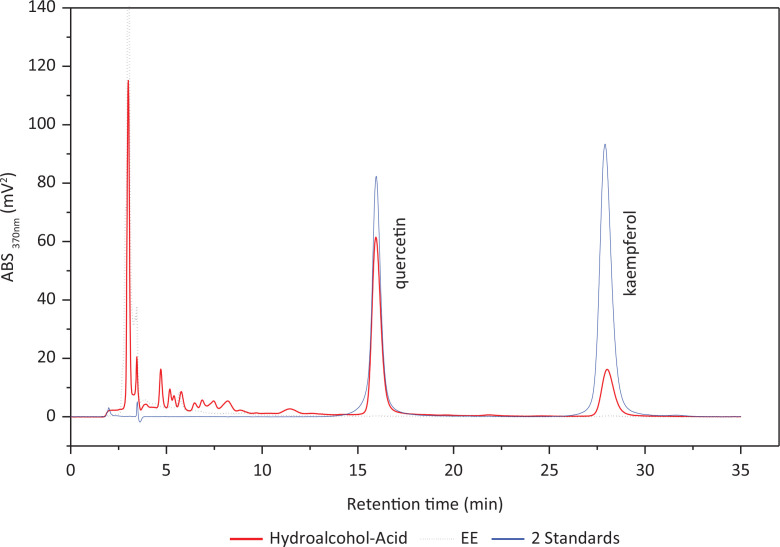
HPLC chromatogram of the standard samples and the ethanol extract of the sericin layer and its hydrolysate.

As our previous research mentioned (28), the hydrolysis-assisted extraction method is specific to the cocoon and is far superior to the colorimetric method. The total flavonoids were estimated through the determination of their aglycones, quercetin, and kaempferol. From Fig. 3, we can see that after hydrolysis, the contents of quercetin and kaempferol increased substantially. The linear regression equations for the two standard samples were as follows: quercetin, *y = −300056.18 + 2074344.21x*, *R*_2_ *= 0.996*; kaempferol *y = −223316.69 + 2816223.82x*, *R*_2_ *= 0.999*. The contents of quercetin and kaempferol were 25.66 ± 0.07 mg/g and 7.76 ± 0.02 mg/g in EE. Therefore, the total flavonoid aglycones were 33.42 ± 0.08 mg/g in EE.

### Antioxidant activity of EE in vitro

**Fig. 4:**
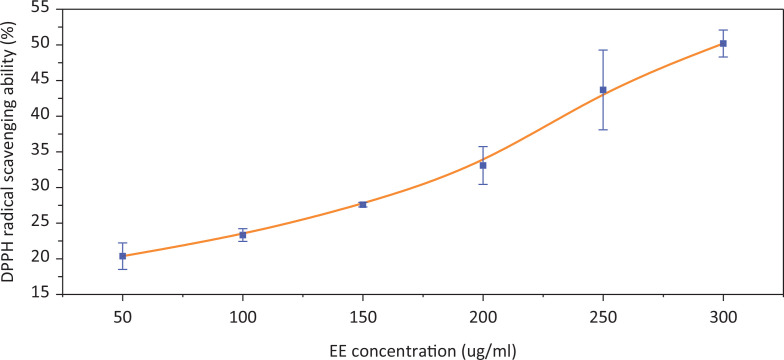
The scavenging effects of EE on DPPH free radicals.

**Fig. 5:**
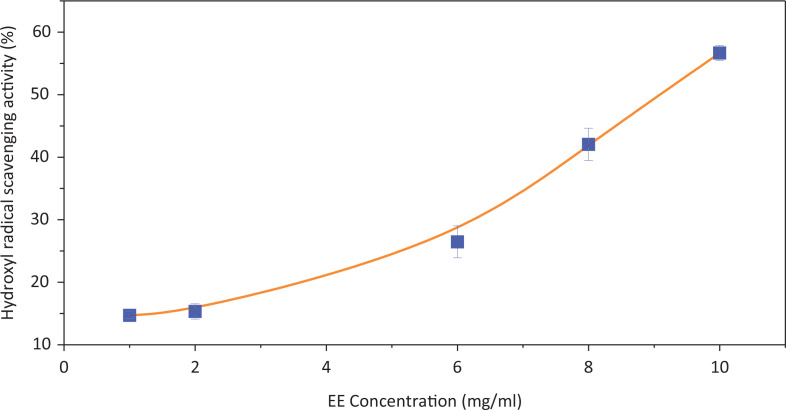
The scavenging effects of EE on ·OH free radicals.

Dietary antioxidants can play a major role in the prevention of oxidation by scavenging free radicals and reducing oxidative stress. DPPH and ·OH radical scavenging activities are the basic methods used to evaluate antioxidant activity *in vitro*. From Figs. 4 and 5, we can see that the DPPH and ·OH radical scavenging activities of EE increased as the concentration of EE increased. The IC_50_ values of EE for DPPH and **·**OH radical scavenging activities were 296.95 ± 13.24 μg/mL and 9.21 ± 0.15 mg/mL. The results indicated that EE showed excellent radical scavenging activity, especially for DPPH.

**Fig. 6:**
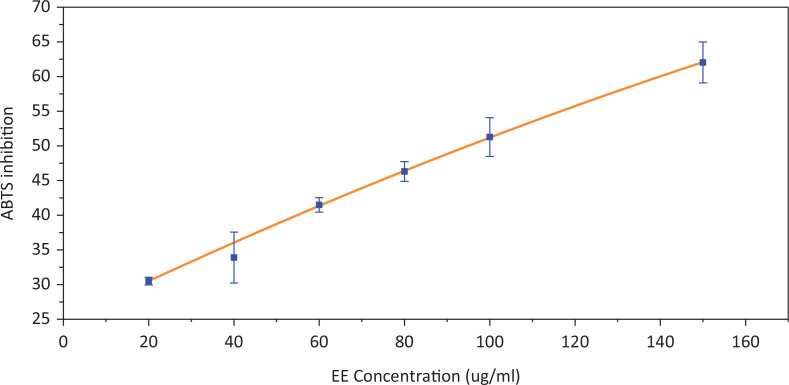
The scavenging effects of EE on ABTS free radicals.

The ABTS assay measured the relative antioxidant ability to scavenge the radical ABTS^+^ and is commonly used to detect the total antioxidant capacity of traditional Chinese medicine components. Fig. 6 shows the scavenging effects of EE on ABTS free radicals. When the EE concentration was 150 μg/mL, the corresponding ABTS inhabitation reached 62%. The IC_50_ value of ABTS scavenging activity was 94.31 ± 9.13 μg/mL. The data indicated that EE could effectively scavenge ABTS radicals.

### α-amylase and α-glucosidase inhibition of EE in vitro

**Fig. 7:**
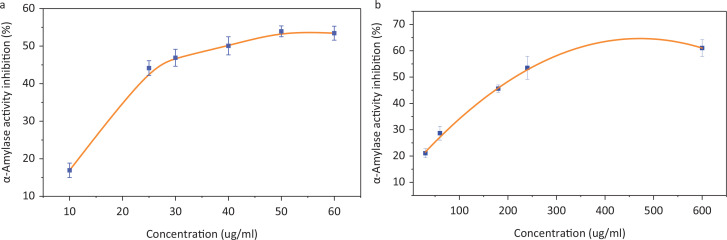
The α-amylase and α-glucosidase activity inhibition of EE. (a) The α-amylase activity inhibition of EE; (b) the α-glucosidase activity inhibition of EE.

The inhibitor of α-amylase and α-glucosidase can impede the hydrolysis and digestion of carbohydrates and reduce the absorption of sugar. The inhibition of EE on α-amylase and α-glucosidase were both dose-dependent (Fig. 7). The inhibition of α-amylase increased rapidly when the concentration of EE was raised from 10 to 25 μg/mL. The IC_50_ value of EE for α-amylase and α-glucosidase inhibition assays were 37.57 ± 6.45 and 212.69 ± 22.94 μg/mL, respectively. The results indicated that EE exhibited potential inhibition of α-amylase and α-glucosidase.

### Effect of EE on the viability of L02 cells

**Fig. 8:**
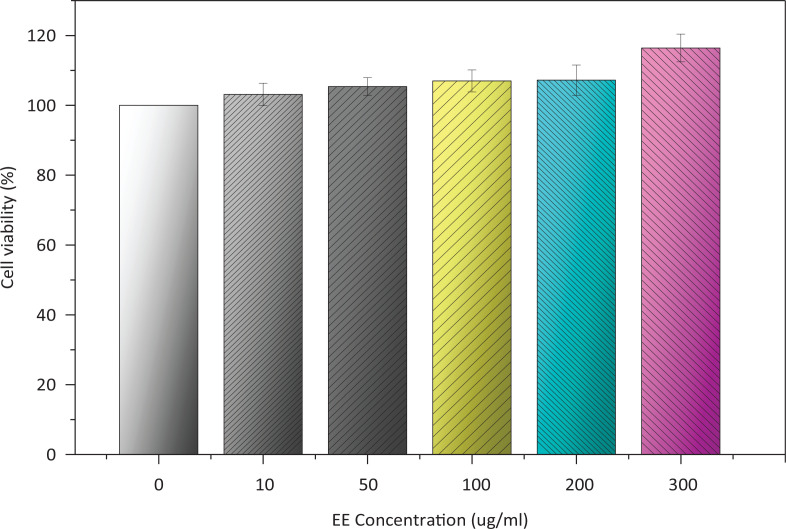
Effect of EE on the viability of L02 cells.

A CCK8 assay was performed to analyse the cytotoxicity of extracts on cells. The concentration range of EE was determined as 10–300 μg/mL (Fig. 8), and the cell viability did not change during EE exposure for 24 h. In addition, 300 μg/mL of EE may enhance cell viability to a certain degree.

### Viability and ROS levels of L02 cultured with high glucose

**Fig. 9:**
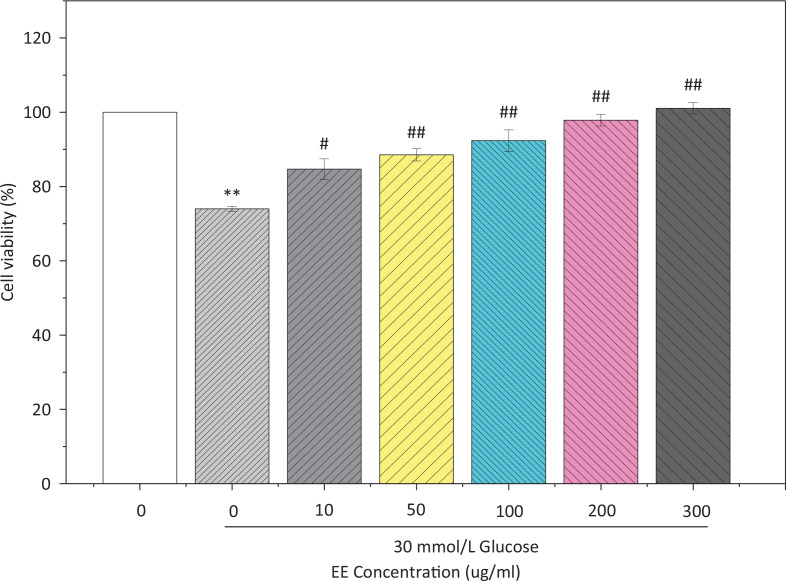
Effect of EE on the viability of L02 cultured with high glucose. ^**^P < 0.01 VS the normal control group; ^#^*P* < 0.05, ^##^*P* < 0.01 VS the model group.

To determine the protective effect of EE against high glucose, L02 cells were treated with 30 mmol/L glucose to simulate a high sugar environment. As shown in Fig. 9, cell viability decreased significantly after glucose treatment. However, the cell viability obviously increased after the treatment of EE, and when the concentration of EE was 200 μg/mL, the cell viability reached 97.86% of the normal group.

**Fig. 10:**
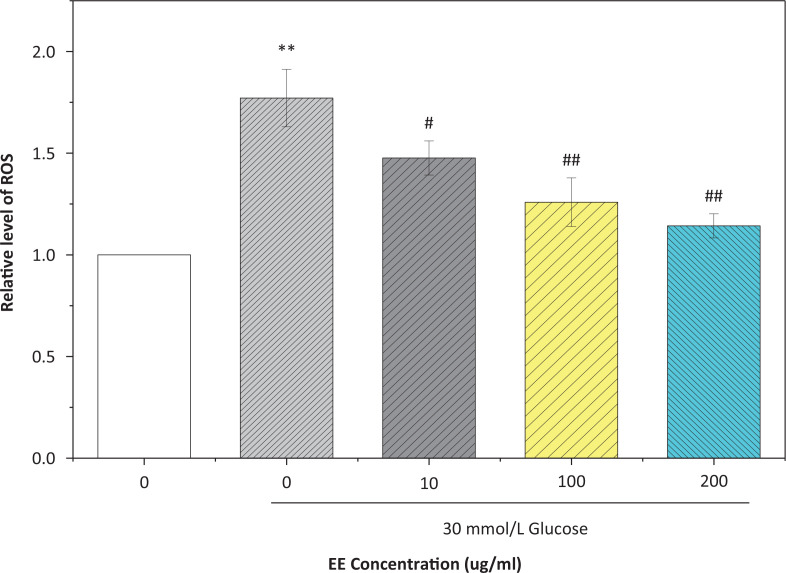
Effect of EE on ROS content in L02 that cultured with high glucose. **P < 0.01 VS the normal control group; #*P* < 0.05, ##*P* < 0.01 VS the model group.

High glucose can increase the level of ROS and cause oxidative stress. The level of ROS in the model group was 1.77 times the level in the normal group (*P* < 0.01). After treatment with different concentrations of EE, the ROS levels decreased significantly (Fig. 10). The ROS level in the 200 μg/mL EE group was close to the normal group, only 1.14 times as much. These results indicated that EE could protect L02 cells and reduce the intracellular oxidative stress induced by high glucose.

### 8-OHdG, NF-κB, and TNF-α content in L02 cells cultured with high glucose

**Fig. 11:**
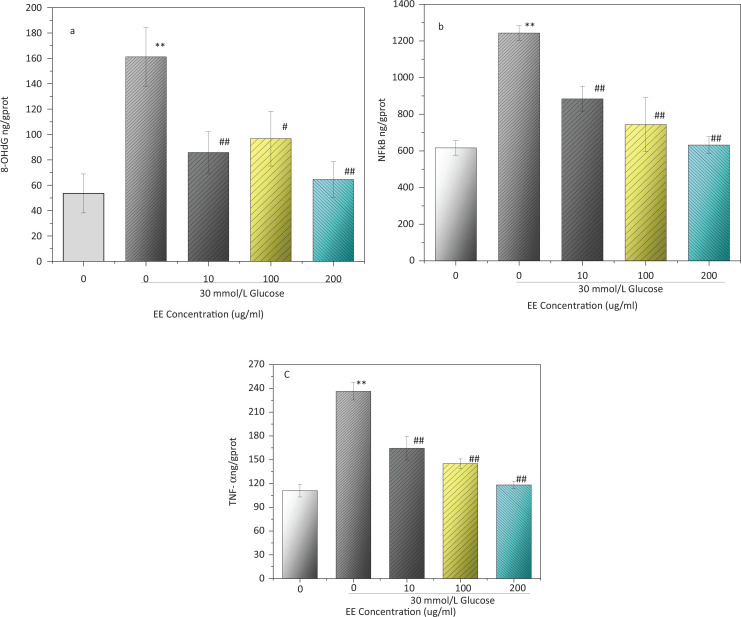
Effect of EE on 8-OHdG (a), NF-κB (b) and TNF-α (c) content in L02 cells cultured with high glucose. ^**^P < 0.01 compared with the normal control group;^#^*P* < 0.05, ^##^*P* < 0.01 compared with the model group.

8-OHdG is a sensitive marker of DNA damage. In Fig. 11a, the level of 8-OHdG (161.21 ± 23.08 ng/gprot) in the model group was significantly higher than that in the normal group (*P* < 0.01). High-glucose treatment can cause oxidative stress in cells, resulting in DNA damage. However, the level of 8-OHdG in the 200 μg/mL EE group decreased to two-thirds of that in the model group. High-glucose treatment also significantly increased the level of NF- κB (*P* < 0.01), reaching 1243.30 ± 40.66 ng/gprot in the model group, which was about double that of the normal group level (Fig. 11b). NF-κB levels decreased to 71.07, 59.81, and 50.82% of that in the model group in the three EE-treatment groups, respectively. TNF-α is a cell signalling protein involved in systemic inflammation and plays an important role in immune regulation and the defence system. The level of TNF-α increased to 236.32 ± 10.87 ng/gprot after high-glucose induction, which was significantly higher than that in the normal group (*P* < 0.01). However, after treatment with three concentrations of EE, the levels of TNF-α dropped to 164.44 ± 16.83 ng/gprot, 145.05 ± 5.86 ng/gprot, and 118.10 ± 4.89 ng/gprot (Fig. 11c). These data indicated that EE may have the potential to alleviate DNA damage and the inflammatory reaction in L02 cells induced by high glucose.

## Discussion

Sericin, a natural macromolecular protein derived from the silkworm *Bombyx mori* has been demonstrated to have a variety of bioactivities. Sericin demonstrated high cells cryoprotective effect (33). It also had the abilities to inhibite tyrosinase and lipid peroxidation (34), suppress mouse skin colon tumorigenesis (35), and reduce oxidative stress (36). In recent decades, the antidiabetic activities of sericin have been developed. Okazaki et al. (25) examined the effect of sericin on the carbohydrate and lipid metabolism in high-fat diet rats. They firstly found that the consumption of sericin remarkably reduced the levels of serum and hepatic lipids in high-fat diet rats. Besides, supplemental sericin could also improve glucose tolerance and elevate the concentration of serum adiponectin in high-fat diet rats. Notably, sericin can not only reduce the blood glucose levels in diabetic rats but also has significant therapeutic effects on treating complications occurring due to diabetes. Intragastrical perfusion of sericin for 35 days could protect sciatic nerve and related nerve cells against diabetes-induced injuries (37).

However, the sources or the procession methods of sericin are usually overlooked. The studies mentioned above often used mixtures of sericin peptides of wide-ranging molecular masses as starting material. The sericin layer has many small amounts of non-sericin ‘impurities’. The non-sericin components mainly contain free amino acids and flavonoids. It is well-known that flavonoids have a wide range of biological activities, including antioxidant (38), hypoglycaemic (39), and anti-tumour (40). Our previous study indicated that the EEs of green cocoon shells (Daizo cocoon) possessed stronger antioxidant activity and inhibitory activity of glucosidase compared to pure sericin *in vitro* (17).

Therefore, we choose a new green cocoon variety (caoyuan × shenyun) which contains abundant flavonoids as material. A hydrolysis-assisted extraction method which is specific to the cocoon and far superior to the colorimetric method was used to analyse the flavonoids in sericin. The flavonoids in extracts of green cocoons are presented in quercetin and kaempferol glycosides. The total amount of the two aglycones was 33.42 ± 0.08 mg/g. Besides, the ethanol extracts showed strong antioxidant activity, as well as α-amylase and α-glucosidase inhibition activities. The results will provide the basis for a better use of sericin.

Chronic hyperglycemia can directly promote the expression of inflammatory factors. The levels of IL-6, IL-18, IL-1, TNF-α, and NF-κB were very high in the blood of diabetic mice (41). Ramesh et al. found that high-glucose environment would cause oxidative damage to DNA in liver and kidney cells. And the level of 8-OHdG, a sensitive DNA damage marker, would increase (42). Our research found that the high glucose treatment caused the increase of 8-OHdG level in L02 cells, while the EE treatment significantly reduced the 8-OHdG level (*P* < 0.01). The 8-OHdG level in 200 g/mL treatment group was even reduced to one-third of it in the model group. This indicates that EE may have the ability to reduce DNA damage induced by high glucose. NF-κB is a key transcription factor that regulates the expression of immune-related genes. It is involved in transcriptional regulation of various cytokines. Over expressed ROS can activate the expression of NF-κB. Activated NF-κB will initiate the transcription of its downstream genes, leading to an overexpression of a range of inflammatory factors including IL-6 and TNF-α (26). Excessive TNF-α can lead to the breakdown of fat particles in fat cells, resulting in an increase of free fatty acid content, which eventually leads to insulin resistance (43). Therefore, the levels of NF-κB and TNF-α in L02 cells were tested in this study. The results showed that EE could significantly inhibit the over expression of NF- κB and TNF-α induced by a high sugar environment.

In summary, a new green cocoon (caoyuan × shenyun) containing high active ingredients was first used to obtain the ethanol extracts here. The extract was composed of amino acids and abundant flavonoids. The ethanol extracts showed strong antioxidant activity, as well as α-amylase and α-glucosidase inhibition activities. In addition, EE had no toxicity to L02 cells and reduced the intracellular oxidative stress in L02 cells induced by high glucose. In addition, EE also alleviated DNA damage and inflammatory reactions in L02 cells induced by high glucose.
